# Carotenoids from Haloarchaea and Their Potential in Biotechnology

**DOI:** 10.3390/md13095508

**Published:** 2015-08-25

**Authors:** Montserrat Rodrigo-Baños, Inés Garbayo, Carlos Vílchez, María José Bonete, Rosa María Martínez-Espinosa

**Affiliations:** 1 Biochemistry and Molecular Biology Division, Agrochemistry and Biochemistry Department, Faculty of Sciences, University of Alicante, Ap. 99, E-03080 Alicante, Spain; E-Mails: rmontserb@gmail.com (M.R.-B.); mjbonete@ua.es (M.J.B.); 2 Algal Biotechnology Group, University of Huelva and Marine International Campus of Excellence (CEIMAR), CIDERTA and Faculty of Sciences, 21071 Huelva, Spain; E-Mails: garbayo@dqcm.uhu.es (I.G.); bital.uhu@gmail.com (C.V.)

**Keywords:** isoprenoid, carotenoids, bacterioruberin, haloarchaea, red and orange pigments

## Abstract

The production of pigments by halophilic archaea has been analysed during the last half a century. The main reasons that sustains this research are: (i) many haloarchaeal species possess high carotenoids production availability; (ii) downstream processes related to carotenoid isolation from haloarchaea is relatively quick, easy and cheap; (iii) carotenoids production by haloarchaea can be improved by genetic modification or even by modifying several cultivation aspects such as nutrition, growth pH, temperature, *etc.*; (iv) carotenoids are needed to support plant and animal life and human well-being; and (v) carotenoids are compounds highly demanded by pharmaceutical, cosmetic and food markets. Several studies about carotenoid production by haloarchaea have been reported so far, most of them focused on pigments isolation or carotenoids production under different culture conditions. However, the understanding of carotenoid metabolism, regulation, and roles of carotenoid derivatives in this group of extreme microorganisms remains mostly unrevealed. The uses of those haloarchaeal pigments have also been poorly explored. This work summarises what has been described so far about carotenoids production by haloarchaea and their potential uses in biotechnology and biomedicine. In particular, new scientific evidence of improved carotenoid production by one of the better known haloarchaeon (*Haloferax mediterranei*) is also discussed.

## 1. Introduction

Carotenoids are pigments that have received considerable attention due to their biotechnological applications and, more importantly, their potential beneficial effects on human health [1,2,3]. These compounds are the second most abundant naturally occurring pigments in nature [4], and they are mainly C_40_ lipophilic isoprenoids ranging from colourless to yellow, orange, and red [5]. The production of such as kind of pigment has been described from plants and some microorganisms such as algae, cyanobacteria, yeast [6] and fungi [7,8].

Plants, algae, yeast, cyanobacteria and fungi have been considered good sources to isolate and even to produce carotenoids at high scale so far [9,10,11,12]. In fact, general characterisations of carotenoids isolated from those organisms are abundant in the literature, in which techniques such as spectrophotometry, thin layer chromatography (TLC), high performance liquid chromatography-mass spectrometry (HPLC-MS) and nuclear magnetic resonance spectroscopy (NMR) are used to define the carotenoids profile from specific species as well as the carotenoids chemical structure [13,14,15,16,17,18]. However, not too much attention has been paid to halophilic microorganisms, and in particular, to haloarchaea as microorganisms with high capability of carotenoids production.

Halophiles comprise a heterogeneous group of microorganisms that require salts for optimal growth. Even high salt concentration up to 4 M is required for some extremophilic species such those belonging the *Haloferacaceae* family, Archaea domain. The pigments produced by these halophilic organisms include phytoene, β-carotene, lycopene, derivatives of bacterioruberin, and salinixanthin [19]. *Dunaliella salina* is one of the better known halophilic microorganisms in terms of carotenoids production [20,21,22,23]. However, apart from that halophilic microalgae, only few studies have been carried out about production of carotenoids by halophiles and in most of the cases, the studies are focused on carotenoids isolation and characterisation by traditional biochemical procedures as those mentioned before [24,25,26].

Within the halophiles there is a family of particular interest in several fields of applications: micro-ecology, biotechnology and extreme metabolic adaptations. This is the case of the *Haloferacaceae* family (previously mentioned) grouping extreme halophilic archaea inhabiting salty environments such as marshes or salty ponds from where NaCl is obtained for human consumption [27,28,29,30]. The first study about carotenoid production by halophilic microorganisms from the *Haloferacaceae* family (previously called *Halobacteriaceae* family*)* were published in the latter half of the 1960s [31,32]. During the last two decades of the last century, several research works demonstrated that some haloarchaeal species not only produce carotenoids but also produce them at high concentration. This fact makes possible to propose haloarchaea as a good natural source for carotenoids production at large scale by means of suitable bioprocess engineering tools, namely specifically designed bioreactors.

This review summarised what it has been described up to now about carotenoids production by haloarchaea and its potential uses in Biotechnology and Biomedicine. The effect of different parameters on carotenogenesis in haloarchaea such as temperature, salt concentration, pH and carbon/nitrogen ration is also discussed.

## 2. Carotenoids: Structure and Functionality

Carotenoids are hydrophobic compounds which essentially consist of a C_40_ hydrocarbon backbone in the case of carotenes (*i.e.*, they contain 40 carbon atoms in eight isoprene residues), often modified by various oxygen-containing functional groups to produce cyclic or acylicxanthophylls. So, all carotenoids possess a long conjugated chain of double bond and a near bilateral symmetry around the central double bond, as common chemical features [33].This chain may be terminated by cyclic groups (rings) and can be complemented with oxygen-containing functional groups [34].

Carotenoids can be classified into different groups on the basis of the criteria used. Based on the basic chemical structure and the oxygen presence, carotenoids are classified into two types: carotenes or carotenoid hydrocarbons, composed of carbon and hydrogen only; and xanthopylls or oxygenated carotenoids, which are oxygenated and may contain epoxy, carbonyl, hydroxyl, methoxy or carboxylic acid functional groups [35]. Lycopene and β-carotene are examples of carotene carotenoids and lutein, canthaxanthin, zeaxanthin, violaxanthin, capsorubin and astaxanthin are xanthopyll carotenoids [36].

The degree of conjugation and the isomerization state of the backbone polyene chromophore determine the absorption properties of each carotenoid. Due to the numerous conjugated double bonds and cyclic end groups, carotenoids present a variety of stereoisomers with different chemical and physical properties. The most important forms commonly found among carotenoids are geometric (*E*-/*Z*-). A double bond links the two residual parts of the molecule either in an *E*-configuration with both parts on opposite sides of the plane, or a *Z*-configuration with both parts on the same side of the plane. Geometrical isomers of this type are inter-convertible in solution. This stereoisomerism exerts a marked influence on the physical properties. The conjugation system described imparts carotenoids with excellent light absorbing properties in the blue-green (450–550 nm) range of the visible spectrum. Because of this reason, biochemical techniques such as UV-Vis spectrophotometry or Raman spectroscopy can be used to analyse carotenoids production by plants and microorganisms [37].

When the criterion used to classify carotenoids is related to vitamin A, then the carotenoids can be categorized as follows: (a) vitamin A precursors that do not pigment such as β-carotene; (b) pigments with partial vitamin A activity such as cryptoxanthin, β-apo-8′-carotenoic acid ethyl ester; (c) non-vitamin A precursors that do not pigment or pigment poorly such as violaxanthin and neoxanthin; and (d) non-vitamin A precursors that pigment such as lutein, zeaxanthin and canthaxanthin [38].

Some of the most important carotenoids in terms of biotechnological and biomedical uses explored so far are: Astaxanthin (3,3′-dihydroxy-β,β′-carotene-4,4′-dione) [36,39,40], β-Carotene (β,β-carotene) [38,41,42,43,44], Canthaxanthin (β,β-carotene-4,4′-dione) [45,46,47,48], β-Cryptoxanthin (hydroxy-β-carotene) [38,49,50,51,52,53,54], Fucoxanthin [38,55], Lycopene (ψ,ψ-carotene) [33,56,57], Lutein (β,ε-carotene-3,3′-diol) [42,58,59,60,61], Zeaxanthin (β,β-carotene-3,3′-diol) [38,62,63], and Violaxanthin (5,6:5′,6′-diepoxy-5,5′,6,6′-tetrahydro-β-carotene-3,3′-diol) [64,65,66,67,68].

## 3. Carotenoids in the Context of Life

Carotenoids have received much attention because of their various and important biological roles in all living systems [4,69,70,71]. Although some of those biological roles have been already mentioned in the previous section, this Section includes details about carotenoids biological roles.

In most of the organisms, the most relevant biological functions of carotenoids are linked to their antioxidant properties, which directly emerge from their molecular structure. Xanthophyll carotenoids in particular are free radical scanvengers, potent quenchers of reactive oxygen species (ROS) and nitrogen oxidative species (NOS), and chain-breaking antioxidants. Asthaxanthin and canthaxantin, for example, are better antioxidants and scanvengers of free radicals than β-carotene. In recent years, the understanding of ROS-induced oxidative stress mechanisms and the search for suitable strategies to fight oxidative stress has become one the major goals of medical research efforts [3]. On the other hand, carotenoid pigments are one of these natural products responsible for colours: yellow, orange, red, and purple colours in a wide variety of plants, animals, and microorganisms are due to those compounds [72].

In animals and humans, these compounds are precursors of vitamin A (provitamin A activity) and retinoid compounds required for morphogenesis and embryonic development [35,73]. Vitamin A is well recognized as a factor of great importance for child health and survival, its deficiency causes disturbances in vision and various related lung, trachea and oral cavity pathologies. Animals and humans cannot synthesize carotenoids *de novo*, although are able to convert them into vitamin A. Diet is the only source for these precursors for retinol synthesis, fruits, vegetables and microalgae being the major suppliers of provitamin A active carotenoids [3,35]. Other biological roles and functions of carotenoids in these organisms include: absorbers of light energy, oxygen transporters, scavengers of active oxygen [2,74], antitumor and enhancers of *in vitro* antibody production [75,76]. In birds and fish, carotenoids are an important signal of good nutritional condition and are used in ornamental displays as a sign of fitness and to increase sexual attractiveness [71,77].

In algae and higher plants, carotenoids serve as regulators of plant growth and development, as accessory pigments in photosynthesis and as a photoprotectors. Thus, they contribute to light harvesting, maintaining the structure and function of photosynthetic complexes, quenching chlorophyll triplet states, scavenging ROS, and dissipating excess energy [34]. On the other hand, carotenoids are also precursors for the hormones abscisic acid (ABA) and strigolactones, and as attractants for other organisms, such as pollinating insects and seed-distributing herbivore [35]. In plants, those pigments are involved in various biological processes, such as photosynthesis, hormones synthesis, photomorphogenesis, photoprotection and development [4]. Apart from these important roles, due to their striking and rich colour, carotenoids are important floral pigments serving to attract pollinators and seed dispersers.

Finally, microorganisms are a great source of diverse carotenoids. As mentioned before for other organisms, in microorganisms, carotenoids are in charge of light protection, cell colour and antioxidative stress mechanisms. It is important to highlight that some carotenoids, such as salinixanthin or thermozeaxanthin, are only produced by some extremophilic microorganisms [78,79]. During the last 30 years, researchers, as well as research and development companies, have paid attention to microorganisms due to the high capability of carotenoid production that some species exhibit. This fact, coupled with new insights on molecular biology techniques and downstream process make those microorganisms good sources for carotenoids production at large scale.

## 4. Carotenoids Metabolism

Carotenoids are derived from the general isoprenoid biosynthetic pathway, along with a variety of other important natural substances such as steroids and gibberellic acid. The starting product required to synthetize all the isoprene derivatives is mevalonic acid which is transformed into a phosphorylated isoprene upon phosphorylation; this isoprene subsequently polymerises. In the course of polymerization, the number and position of the double bonds are fixed.

The synthesis and degradation of carotenes and xanthophylls, the regulation of carotenogenesis, as well as the role of these compounds, have been very well described in plants [4,80] and mammals [81]. Multi-gene engineering approaches have also contributed to better understanding of carotenoid metabolism [82].

The conversion of two molecules of geranylgeranyl pyrophosphate (GGPP) to phytoene, a compound common to all C_40_ carotenogenic organisms, constitutes the first reaction unique to the carotenoid branch of isoprenoid metabolism. From this step, slightly different reactions can be found in different organisms. Anoxygenic photosynthetic bacteria, non photosynthetic bacteria, and fungi desaturate phytoene either three or four times to yield neurosporene or lycopene, respectively. In contrast, oxygenic photosynthetic organisms (cyanobacteria, algae, and higher plants) convert phytoene to lycopene via carotene in two distinct sets of reactions. At the level of neurosporene or lycopene, the carotenoid biosynthesis pathways of different organism’s branch to generate the huge diversity of carotenoids found in nature.

In photosynthetic organisms and tissues, the lipophilic carotenoid and bacteriochlorophyll (Bchl) or chlorophyll (Chl) pigment molecules associate non-covalently but specifically with integral membrane proteins. In non photosynthetic organisms and tissues, carotenoids, often protein bound, occur in cytoplasmic or cell wall membranes, oil droplets, crystals, and fibrils.

As mentioned before, animals are not able to synthesise carotenoids *de novo*. They are acquired throughout the diet. In human beings, it has been well demonstrated that most of the ingested carotenoids are absorbed into the gastrointestinal mucosal cells and appear unchanged in the circulation and tissues. In the intestine, the carotenoids are absorbed by passive diffusion after being incorporated into the micelles that are formed by dietary fat and bile acids. The micellar carotenoids are then incorporated into the chylomicrons and released into the lymphatic system [33]. Carotenoids are transported in the plasma exclusively by lipoproteins. Oxygen-functionalized carotenoids are more polar than carotenes. Thus, α-carotene, β-carotene and lycopene tend to predominate in low-density lipoproteins (LDL) in the circulation, whereas high-density lipoproteins (HDL) are major carriers of xanthophylls, such as cryptoxanthins, lutein and zeaxanthin. The delivery of carotenoids to extrahepatic tissues is accomplished through the interaction of lipoprotein particles with receptors and the degradation by lipoprotein lipase [83,84].

Although no less than forty carotenoids are usually ingested in the diet, only six carotenoids and their metabolites have been found in human tissues, suggesting selectivity in the intestinal absorption of carotenoids. In contrast, thirty-four carotenoids and eight metabolites are detected in breast milk and serum of lactating mothers. Recently, facilitated diffusion in addition to simple diffusion has been reported to mediate the intestinal absorption of carotenoids in mammals. The selective absorption of carotenoids may be due to uptake to the intestinal epithelia by means of facilitated diffusion and an unknown mechanism of excretion into the intestinal lumen. It is well known that β-carotene can be metabolised to vitamin A after intestinal absorption of carotenoids, but little is known about the metabolic transformation of non-provitamin A xanthophylls. The enzymatic oxidation of the secondary hydroxyl group leading to keto-carotenoids would occur as a common pathway of xanthophyll metabolism in mammals [38].

## 5. Production of Carotenoids by Haloarchaea

### 5.1. Type, Content and Biosynthesis of Haloarchaeal Carotenoids

Halophilic archaea are extreme halophilic microorganisms mainly grouped into the *Haloferacaceae* family, phylum *Euryarchaeota*, *Archaea* domain. They are (mostly) aerobic and generally red-pigmented. They constitute the predominant microbial communities in extreme halophilic environments as it was mentioned before. To be alive under those conditions they have adopted several strategies: (i) amino acidic residues predominate in halophilic proteins surface; (ii) cells accumulate high KCl intracellular concentrations to deal with high ionic strength or some osmolytes such as 2-sulfotrehalose [85]; (ii) cellular bilayers have different composition and structure, *etc.* Due to these adaptations, haloarchaea have become a good and innovative source of different molecules of high interest in biotechnology such as enzymes able to be active at high temperature and high ionic strength [86,87], PHB and PHA, carotenoids, *etc.*

**Figure 1:**
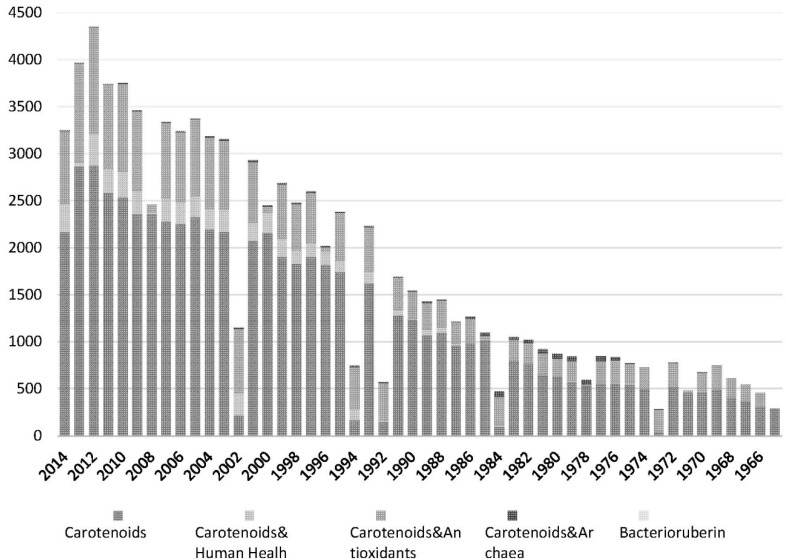
Bars plot summarizing details about the number of publications per year related to carotenoids. The key words used to perform the search were: carotenoids, carotenoids & human health, carotenoids & antioxidants; carotenoids & archaea and bacterioruberin. Pubmed and Scopus were used as databases to do the search.

Related to carotenoids, there is little information in the literature about the carotenoid profile of extremophile microorganisms compared with the information available from other organisms, and only few of them are focused on carotenoid production by archaea in general, and by haloarchaea in particular [58]. Figure 1 summarises the number of publications focused on carotenoids. It is important to highlight that despite the huge number of publications on that subject, only 1.3% of them are related to haloarchaeal carotenoids (780 papers about haloarchaeal carotenoids *vs.* 61590 papers about carotenoids in general). What is clearly supported by the literature is that most members of the family *Haloferacaceae* can synthesize C_50_ carotenoids, including bacterioruberin (as the most abundant C_50_ in most of the analysed haloarchaeal species) and its precursors (2-isopentenyl-3,4-dehydrorhodopin (IDR), bisanhydrobacterioruberin (BABR), and monoanhydrobacterioruberin(MABR)) [32,88]. Several other derivatives have been found in minor amounts, such as 3,4-dehidromonoanhydrobacterioruberin, haloxanthin (which is a derivative of the previous one containing a peroxide end group) and 3,4-epoxymonoanhydrobacterioruberin, identified in *Haloferax volcanii* [26,89]. Other carotenoids such as phytoene, lycopene, and β-carotene are also produced by these species but at lower concentration [7]. Those carotenoids are located in the cell membrane and they are in charge of the colour shown by the red colonies when haloarchaea cells grow on solid media or the red colour shown by salted coastal ponds (mainly in summer). In fact, the content of bacterioruberin pigments in the biomass has been used to monitor the density of halophilic archaeal communities in halophilic environments [90].

Other carotenoids have been identified at very low concentrations in halophilic archaea: lycopersene, *cis*- and *trans*-phytoene, *cis*- and *trans*-phytofluene, neo-β-carotene and neo-α-carotene. The low concentrations of these compounds suggest that they may be used as precursors for the synthesis of other carotenoids including lycopene, retinal and the members of the bacterioruberin group. Some species may also produce the ketocarotenoid canthaxanthin in addition to other carotenoids [58]. Although this is the general carotenoid profile exhibited for most of the haloarchaeal species, it is important to note that some of them can produce high amounts of canthanxanthin, β-carotene and *trans*-astaxantin [91].

The presence of characteristic carotenoids (α-bacterioruberin and derivatives) in haloarchaea cells is easy to identify by Raman spectroscopy [30,92,93]. Thanks to this technique, α-bacterioruberin has been identified as the mayor carotenoid in the following haloarchaea: *Halobacterium salinarum* strains NRC-1 and R1, *Halorubrum sodomense*, *Haloarcula vallismortis* [78] and *Haloarcula japonica* (68.1% of the total carotenoids (mol %) [5]*.* The last species is also able to produce monoanhydrobacterioruberin (22.5%), bisanhydrobactrioruberin (9.3%), and isopentenyldehidrorhodopin (<0.1%) [5]. The main carotenoids produced by *Halorubrum* sp. TBZ126 were bacterioruberin, lycopene and β-carotene [58,79], while the major carotenoid produced by *Halococcus morrhuae* and *Halobacterium salinarum* was all-*trans*-bacterioruberin, accounting for 69% of the carotenoids, respectively [79].

Ronnekleivand colleagues [26] reported that *Haloferax volcanii* contained the (2*S*,2′*S*)-bacterioruberin (82% of total carotenoid), monoanydrobacterioruberin (7%), (2*S*,2ʹ*S*)-bisanhydrobacterioruberin (3%), 3,4-dihydromonoanhydrobacterioruberin (2%) and two undecaene C_50_H_74_O_4_ carotenoids (each 2%), the C_45_-carotenoid (2*S*)-2-isopentenyl-3,4-dehydrorhodopin (1%) and lycopene (0.3%). The lipid composition of the extremely halophilic archaeon *Haloquadratum walsbyi* was investigated by thin layer chromatography and electrospray ionization-mass spectrometry. The results confirmed the presence of the carotenoids carotene and bacterioruberin, the C_30_-isoprenoid compound squalene and the menaquinone with eight isoprenoid units vitamin MK-8 [94].

The total carotenoid content in *Haloarcula japonica* was 335 μg·g^−1^ of dry mass, although the contents in *Halobacterium salinarum* and *Halococcus morrhuae* were 89 and 45 μg·g^−1^, respectively [79].

Although general knowledge about carotenoids biosynthesis and their assimilation in higher plants and human beings is considerable, nutritional functions, as well as metabolic pathways and their regulation, have not been examined in detail in haloarchaea [38]. The biosynthesis of carotenoids in haloarchaea was studied for the first time in the later 1970s. At that time, it was stated that the biosynthetic pathway for the formation of C_40_ carotenes in *Halobacterium* proceeds as follows: isopentenyl pyrophosphate leads to *trans*-phytoene, leads to *trans*-phytofluene, leads to ζ-carotene, leads to neurosporene, leads to lycopene, leads to gamma-carotene, and finally leads to β-carotene. This pathway differs from that in higher plants in that the *cis* isomers of phytoene and phytofluene are not on the main pathway of carotene biosynthesis, as they are in higher plants [95]. On the other hand, it has been suggested that bacterioruberin is synthesised by addition of C_5_ isoprene units to each end of the lycopene chain, followed by the introduction of four hydroxyl groups. The evidence supporting these suggestions where reported around 40 years ago from experiments where nicotine was used to inhibit the bacterioruberin synthesis [96,97]. The presence of multiple genes for several steps in *Halobacterium* NRC-1 carotenoid production suggests that there may be more than one biosynthetic pathway [98,99].

**Figure 2:**
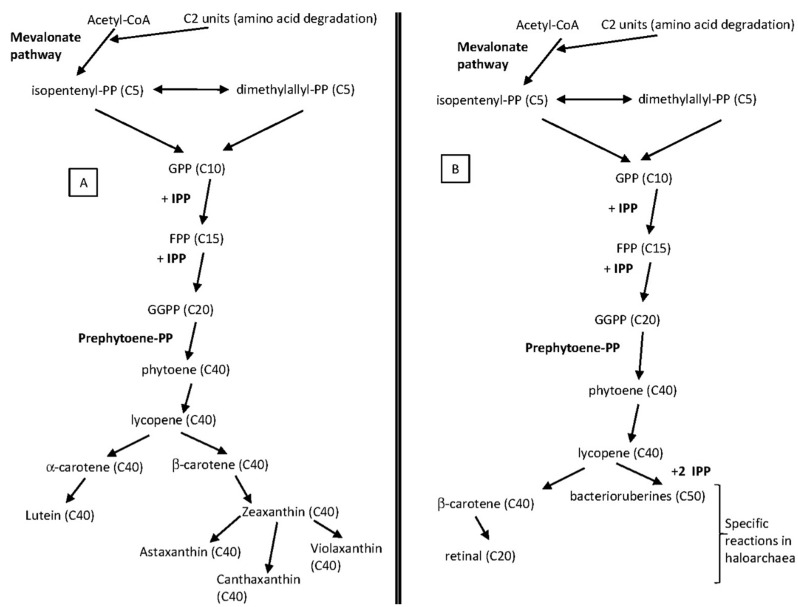
Comparison between the biosynthesis of isoprenoids in photosynthetic organisms (**A**) and the biosynthesis pathway proposed in haloarchaea (**B**). C5 prenyl units are synthesized via the mevalonate pathway starting from two acetyl-CoA molecules and C2 unit arising from amino acid degradation. *Cis*- and *trans*-prenyl chains are derived through head-tail (HT) condensation steps with isopentenyl-diphosphate (IPP). C15 and C20 prenyl chains are modified by head-head condensations and desaturase reactions. Genes coding for the enzymes catalysing those reactions have been identified in the *Halobacterium salinarum* genome. β-Carotene is the precursor for retinal synthesis while lycopene is the precursor for bacterioruberines in the pathway proposed from the *Halobacterium salinarum*’s genomic analysis [99]. Preliminary evidence from other genomic analysis (*Haloferax* sp.) also support this proposal [104]. Details about genes, enzymes and chemical reactions involved in retinal and bacterioruberines synthesis are far from known. There are not reports about the biosynthesis reactions of other carotenoids such as zeaxanthin, canthaxanthin, astaxanthin, *etc*. in haloarchaea. GPP = geranyl diphosphate; FPP = farnesyl diphosphate; GGPP = geranylgeranyl diphosphate.

Computational genome and pathway analysis of halophilic Archaea done by Falb and co-workers [100] suggested that phytoene is reduced to lycopene by phytoene desaturase. Lycopene is the branching point for the synthesis of bacterioruberins (C_50_) and β-carotene (C_40_) [101,102]. Although the reactions leading from lycopene to bacterioruberins have not been elucidated in detail yet, there is some evidence supporting that the lycopene cyclase (OE3983R) converts lycopene to β-carotene in *Halobacterium salinarum* str. NRC-1 [98]. More recently, studies carried out in *Haloarcula japonica* have clearly identified that the genes named *c0507*, *c0506*, and *c0505* encoded a carotenoid 3,4-desaturase (CrtD), a bifunctional lycopene elongase and 1,2-hydratase (LyeJ), and a C_50_ carotenoid 2″,3″-hydratase (CruF), respectively. The above three carotenoid biosynthetic enzymes catalyse the reactions that convert lycopene to bacterioruberin in *Haloarcula japonica* [103]. Figure 2 compares the biosynthesis of carotenoids in photosynthetic organisms and in haloarchaea (on the basis of the results reported from *Halobacterium*, *Haloarcula* and preliminary evidence from *Haloferax* genomic analysis).

### 5.2. Bacterioruberin Is One of the Major Carotenoids Produced by Haloarchaea

As it can be concluded from the previous section, bacterioruberin is the main carotenoid component responsible for the colour of the red archaea of the family *Halobacteriaceae*. This pigment has a rather different molecular structure. It has a primary conjugated isoprenoid chain length of 13 C=C units with no subsidiary conjugation arising from terminal groups, which contain four –OH group functionalities only [37,78]. Table 1 summarises the bacterioruberin chemical structure as well as its derivatives.

**Table 1:** Chemical structures of bacterioruberin and its derivatives [79,105].

Name	Chemical Structure
Bacterioruberin	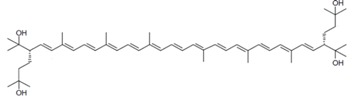
Monoanhydrobacterioruberin	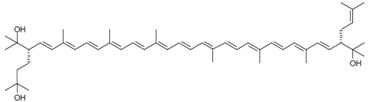
Bisanhydrobacterioruberin	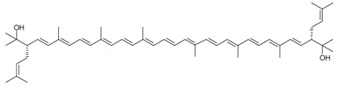
Trisanhydrobacterioruberin	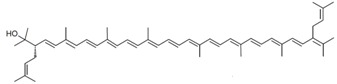
2-isopentenyl-3,4-dehydrorhodopin	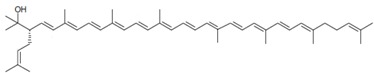
5-*cis*-bacterioruberin	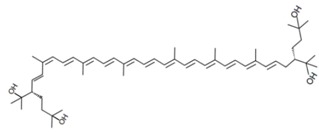
9-*cis*-bacterioruberin	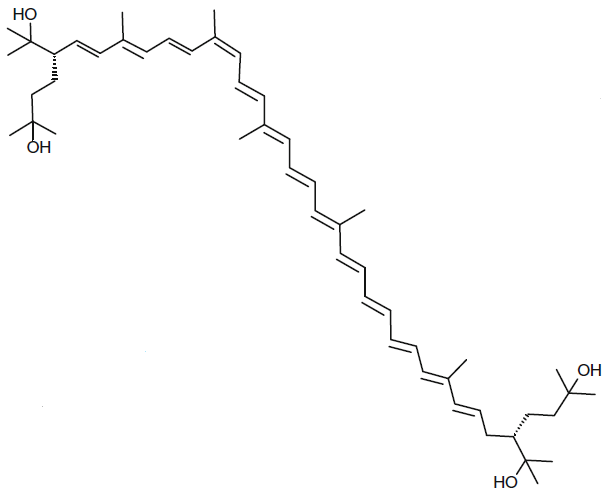
13-*cis*-bacterioruberin	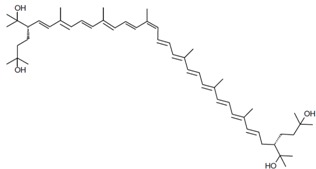

**Figure 3:**
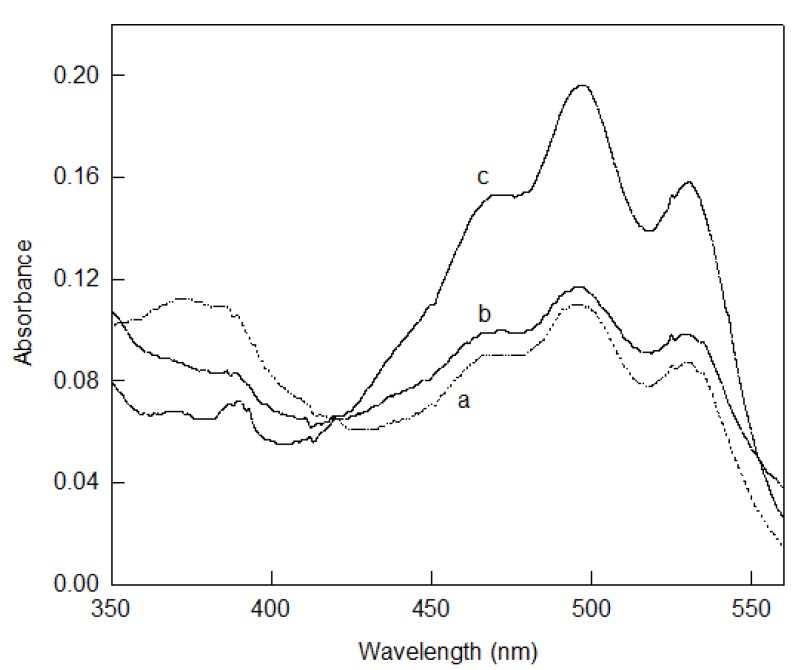
Absorption spectra of acetone extracts of *Haloferax mediterranei* cells grown in complex medium (**a**) pH 5; (**b**) pH 7 and (**c**) pH 9. *Hfx. mediterranei* was grown in complex medium pH 7 (0.5% yeast extract and 25% salted water) until shortly before the culture entered the stationary phase, after which cells were transferred to fresh complex medium pH 5, 7 or 9.

This pigment protects the cells against damage produced by high intensities of light in the visible and ultraviolet range of the spectrum and provides aid in photoreactivation [106,107]. It is also involved in the reinforcement of the cell membrane. It was described for the first time from cells of *Halobacterium* species [88,108,109]. The byosinthesis of C_50_ carotenoids in general terms, and the effect of several chemical compounds on this biosynthesis were first described from *Halobacterium cutirubrum* (*Halobacteriaceae* family) [88,97,110]. A few years later, it was described that bacterioruberin is synthesized from other C_50_ carotenoids, such as isopentenyldehydrorhodopin, bisanhydrobacterioruberin, and monoanhydrobacterioruberin [5] and the synthesis is induced by (i) low oxygen tension and high light intensity [111,112]; (ii) osmotic stress [113]; and (iii) the presence of different compounds such as aniline [114] (Figure 2). However, this general pattern has some exceptions, for *example Haloquadratum walsbyi*: cells grown under osmotic stress did not experience changes in terms of either membrane lipid composition or carotenoids production [94]. Composition of the total carotenoids fraction in haloarchaea can also change on the basis of the nutritive factors within the culture media [105], the light intensity, oxygen tension, NaCl concentration [91,105,111], and other physical-chemical parameters such as pH value of the culture media. Figure 3 shows the effect of pH on carotenoids profile in *Haloferax mediterranei* cells grown in aerobic complex media. It has been reported that pH significantly influences cell growth and total carotenoid production in a lot of microorganism [115]. Hamidi *et al.* reported an analysis of pH and other environmental factors through response surface methodology on the total carotenoid production of extremely halophilic archaeon *Halorubrum* sp. TBZ126 [116]. They have found that optimum conditions for biomass and total carotenoid production occurred between pH 7 and 10 and biomass and total carotenoid production in pH 10 was about 93% and 90% respectively, compared to data reached from optimum conditions.

More recently, bacterioruberin has been used for the detection of extremely halophilic archaea embedded in halite in terrestrial and possibly extra-terrestrial samples [117].

Although bacterioruberin and its derivatives possess extraordinary biological functions, the research regarding the biosynthesis regulation or practical applications of C_50_ carotenoids produced by halophilic archaea is still scarce.

### 5.3. Bacterioruberin Biological Roles

*Bacterioruberin as antioxidant compound:* The scavenging capacity of the oxygen reactive species (ROS) is dependent on the carotenoid concentration as it has been described so far. On the other hand, the antioxidant capacity of carotenoids in general is related to the length of the carbon chain, the number of pairs of conjugated double bonds and the carotenoids concentration [118,119,120]. As mentioned before, bacterioruberin contains 13 pairs of conjugated double bonds *versus* the nine pairs of conjugated double bonds of the β-carotene. Therefore, bacterioruberin is much better than β-carotene as radical scavenger [5,121]. It has been demonstrated that it protects the cells against oxidative damage. As a consequence of this important biological role, haloarchaea escape from fatal injury under strong light, and resist oxidative DNA damage resulting from radiography, UV irradiation, high doses (5 kGy) of gamma irradiation and H_2_O_2_ exposure [107,122]. What is clearly stated up to now is that the carotenoids of halophilic microorganisms present higher antioxidant capacity than the carotenoids produced by the other microorganism (extremophilic or not extremophilic).

*Bacterioruberin controls membrane rigidity:* With 4 hydroxyl substitutes in this dipolar C_50_ carotenoid, bacterioruberin was suggested to act as a “rivet” in the membrane cells. This carotenoid has some effect on fluidity of the membrane, acts as a barrier to water and allows permeability to oxygen and other molecules, so strains can survive in hypersaline or low-temperature conditions [105,123].

*Bacterioruberin as part of the rhodopsin complexes:* Archaerhodopsin-2 (aR2) is a retinal protein-carotenoid complex found in the claret membrane of *Halorubrum* sp. as well as in other species [124,125,126]. It functions as a light-driven proton pump highly important for haloarchaea cells to obtain energy. Using crystallographic studies it has been demonstrated that bacterioruberin binds to crevices between the subunits of the archaerhodopsin structure, which is a trimer. So, bacterioruberin sustains structural support related to the archaerhodopsin structure [127]. Bacterioruberin is also part of a complex constituted by this carotenoid and halorhodopsin in haloarchaea membranes such as those from *Natronomonas pharaonis.* Halorhodopsin is a retinal protein with a seven-transmembrane helix and acts as an inward light-driven Cl(−) pump [128].

## 6. Biotechnological Uses and Production Potentiality of Carotenoids from Haloarchaea

### 6.1. Biotechnological Uses of Carotenoids from Haloarchaea

Carotenoids have numerous applications as colorants (in food products and cosmetics), feed additives for poultry, livestock, fish, and crustaceans (Patent ES2324077 A1. See Table 2), antioxidants, antitumor and heart disease prevention agents, precursors of vitamin A and enhancers of *in vitro* antibody production. Hence, they are widely applied in the food, medical, pharmaceutical, and cosmetic industries as dyes and functional ingredients [3,6,58].

**Table 2:** Patents (last 20 years) related to carotenoids production by haloarchaea or its biotechnological uses (as pigments or as antioxidants). The key words used to find out the patents were: bacterioruberin, halobacteria, haloarchaea and carotenoids. Data obtained from different websites [129,130,131,132,133,134,135].

Publication Number	Publication Date	Title	International Application Number
WO/2009/042734	02.04.2009	Radiation-resistant mutants of a halophilic archaeon and uses thereof	PCT/US2008/077596
ES2324077 A1	29.07.2009	Compuesto a base de membranas celulares liofilizadas	
US 7939220 B2	10.05.2011	Proton-translocating retinal protein	PCT/EP2001/008715
WO2011133907 A2	27.10.2011	Methods to increase and harvest desired metabolite production in algae	PCT/US2011/033637
WO2012169623	13.12.2012	Method for producing carotenoid each having 50 carbon atoms	PCT/JP2012/064817
WO2014045280 A1	27.03.2014	Topical halobacteria extract composition for treating radiation skin tissue damage	PCT/IL2013/050786
WO/2014/045279	27.03.2014	Halobacteria extracts composition for tumour reduction	PCT/IL2013/050785
US 20140356854 A1	4.10.2014	Methods and compositions relating to mevalonate phosphate decarboxylase	
07-132096	23.05.1995	Production of C_50_ Carotenoid	

In some of the patents, the authors use the term Halobacteria, which was the first name used to identify what it is now call haloarchaea (Families *Halobacteriaceae* and *Haloferacaceae*).).

More than 600 carotenoids are known to occur naturally and many of them are still being identified. Although the carotenoids’ market is highly segmented it has grown significantly in the last few years and this growth is projected to continue. β-Carotene and the xanthophylls astaxanthin, cantaxanthin, and lutein are the major carotenoids with commercial interest, and Europe is currently the largest market for this kind of compounds with nearly 45% of worldwide sales [136]. There are several advantages and disadvantages of chemical synthesis for carotenoids production. Chemical synthesis technology has been developed so far for many carotenoids (mainly for all of those most demanded by the market). This synthesis procedures produce carotenoids of exceptional purity and consistency at high concentration, and usually the overall cost of the production is relatively low. However, the chemical synthesis of certain carotenoids is very complex, and as a consequence of that, it is slow and expensive. Besides, the chemical synthesis of a new carotenoid usually requires the development of a new chemical route *in vitro*. Finally, some stereoisomers may not be active as the naturally occurring carotenoids isomers, or may have undesired side effects. As a consequence of all those aspects, the production of carotenoids from biological sources has been an area of intensive investigation. Also the consumer preference for natural products, as well as high costs, presence of by-products and damaging effects on the environment have together intensified efforts to identify alternative sources for chemical method. The production of natural colorants through fermentation has a number of advantages, such as cheaper production, higher yields, possibly easier extraction, less batch-to-batch variations and no seasonal variations. The production is flexible and can easily be controlled. Furthermore, the collection of microbial organisms is sustainable and usually microbial engineering has no negative impact on the environment [58]. Accordingly for all the previous reasons, different species of bacteria, moulds, yeasts and algae have attracted a great interest as alternative biosources for high-scale production of carotenoids [137,138]. Carotenoid-producing microorganisms have biotechnological attributes proper of microbial cells (fast growth in liquid culture and ability to accumulate or secrete some metabolites). Besides, the use of several molecular biology techniques enables the production of mutant strains able to overproduce carotenoids of interest. For these reasons, microorganisms have become excellent tools to look for new applied processes to obtain biomolecules of high interest in biotechnology and biomedicine and represent the basis of biotechnology-based companies [34].

New research results highlight the possibility of using halophilic microorganisms (mainly halophilic archaea) as natural sources for carotenoids production [19] thanks to the simplicity in increasing carotenoid production by culture conditions and genetic manipulation, and the feasibility of downstream processes of the cells to isolate the carotenoids. The extremely halophilic archaea has unique features making them suitable potential sources for carotenoids production, including: (i) the high-salt tolerance of haloarchaea enables their cultivation under non-sterile conditions because high salt concentrations prevent contamination by other organisms. This feature makes cultivation of haloarchaea advantageous if compared to cultivation of other microorganisms; (ii) the process to obtain the carotenoids is simple because in lower NaCl concentrations cell lysis is induced and consequently, carotenoids extraction could be conducted directly from the cells without any mechanical operation which is required in case of plants and (iii) the procedures for pigments extraction and purification seem to be simpler than those from other sources. Therefore, production potentiality of carotenoids from halophilic haloarchaea should be studied in order to assess alternative commercial sources for carotenoids [58].

### 6.2. Production Potentiality of Carotenoids from Haloarchaea

There are few examples of studies about haloarchaea carotenoids accumulation supporting the idea that these microorganisms might be considered good carotenoids producers [113,137], specifically for bacterioruberin and its C_50_-related pigments. The culture conditions that are required to promote fast growth of halophilic archaea include high salt concentration (from 20% to 25% w/v). However, promoting massive carotenoid accumulation of these halophilic microorganisms generally requires much lower concentrations or NaCl, normally below 16% w/v [113,116]. Such lower salt concentrations address slower growth rates or even cell lysis. Therefore, carotenoid accumulation and growth of halophilic archaea are often opposite events. Moreover, besides the culture medium salinity, other key factors as temperature and pH may affect carotenoids accumulation and also growth rates of halophilic microorganisms tremendously [116]. In some cases, changes in the nutrient composition of the culture medium might result in enhanced C_50_ carotenoid accumulation [105]. Consequently, if cultivated under suitable conditions halophilic microorganisms may accumulate carotenoids.

So far, only few studies have been reported on the accumulation of carotenoids in halophilic archaea, and all of them at laboratory scale systems. The data obtained so far help clarify that the carotenoid accumulation potential of halophilic archaea is worth being studied in terms of bioprocess engineering. The maximal intracellular concentration of carotenoids reported so far for halophilic archaea were obtained in small flasks during laboratory experiments and ranges from 20 to 25 mg·g^−1^ dry weight, with maximal volumetric productions of about 10 mg·L^−1^ [116]. The intracellular concentration of 20–25 mg·g^−1^ dry weight means an accumulation rate per biomass unit of 2.0%–2.5%. These data compare well to those of carotenoid producing microalgae, many of which are below 1% [139] per biomass unit with the exception of *Dunaliella salina*, obviously the most efficient carotenoid producer microorganism [140].

Productivity is the key parameter to understand the potential of a microorganism for production of whatever value compound. For biomass, volumetric productivity is calculated in terms of g·L^−1^·day^−1^, and productivity of a desirable compound can also be calculated per reactor volume (g·L^−1^·day^−1^) or as mg·g^−1^ dry weight·day^−1^. At large scale, surface is included as a factor to which productivity is referred. For instance, biomass areal productivity is expressed as g·m^−2^·day^−1^. Independently of how productivity is expressed, the rate at which biomass is produced in time obviously determines the potential of the microorganism for accumulation of desirable compounds. To our knowledge, no data have been published on both biomass and carotenoid productivities of halophilic archaea at scales other than just few examples in laboratory flasks. Data such these are needed to approach the potential of halophilic archaea for carotenoids production. An approach to the biomass productivity of halophilic archaea can be done simply with the data above. For instance, the average time taken for a culture of *Halorubrum* sp. and other halophilic archaea species to grow to the end of the exponential phase is about 10 days [116,141], in batch systems. Maximal biomass concentration reported is about 0.8 g·L^−1^. Simple calculations give an average biomass productivity of 0.08 g (dry biomass)·L^−1^·day^−1^. This productivity data should be higher if the halophilic archaea were cultured in continuous production systems at optimal growing conditions. Accordingly, there should still be significant room for improving such laboratory biomass productivity data. Combining the average biomass productivity data (0.08 g (dry biomass)·L^−1^·day^−1^) and the maximal intracellular concentration of carotenoids found for *Halorubrum* sp. under specific conditions, 25 mg·g^−1^ dry biomass, a maximal productivity of carotenoids of about 2 mg·L^−1^·day^−1^ is obtained. Microalgae, natural carotenoid producers, can be produced at biomass volumetric productivities likely between 0.1 and 0.3 g·L^−1^·day^−1^ [139,142], which means maximal carotenoids productivity of about 1 to 3 mg·L^−1^·day^−1^ for a microalga that accumulates carotenoids at 1% (w/w), therefore giving haloarchaea a chance to be considered in studies for assessing potential of carotenoid production. Of course, these productivity data are far from those carotenoid productivities obtained with *Dunaliella* species, which are 5 to 10-fold higher. Microalgae have the advantage of using natural light as energy source and carbon dioxide, and haloarchaea, which do not need light to grow, should have the advantage of being produced at a larger volume per surface ratio; this might help to overcome lower volumetric biomass productivities.

These aspects might at least be promising enough to study continuous processes of carotenoids production from halophilic archaea in laboratory and pre-pilot scale, particularly for production of other carotenoids than those typically obtained from microalgae (C_50_-bacterioruberin and derivate C_50_ pigments). Therefore, it is suggested that haloarchaea might become complementary to those already known to be good carotenoid producers, namely microalgae, in the panel of potential producers. Of course this should just be the starting point of a challenging subject. The use of cheap, raw suitable carbon sources, the economics of cultivation—particularly energy costs for mixing and harvesting—biomass processing and carotenoid purification costs are among those key factors that should be extensively studied.

Although the biological roles of the carotenoids produced by haloarchaea in haloarchaeal cells are known (see Section 5), potential benefits of those carotenoids on animal cells (including human beings) have not been tested yet. However, there is some evidence supporting that carotenoids from haloarchaea are, at least, as efficient as those antioxidant compounds produced by other microorganism [79,143]. For instance, the halophilic bacteria *Halobacterium salinarum* produces various pigments such as phytoene, β-carotene, lycopene and derivatives of bacterioruberin and salinixanthin. These pigments have been tested for their free radical scavenging activity by DPPH (di(phenyl)-(2,4,6-trinitrophenyl)iminoazanium) assay and the results validated the known antioxidant activity of carotenoids. A further analysis of the cytotoxic properties against human liver cancer cell lines showed dose-dependent increase in cytotoxicity of the carotenoids on these cells, suggesting the probable anti-cancer properties [143,144]. This is the reason why several research groups around the world as well as some I&D (innovation and development) companies focused on secondary metabolites production have focused their attention on haloarchaea as carotenoids source. This interest is not only supported by the huge amount of publications on that subject, but also by the patents related to carotenoid production by haloarchaea (wild types as well as genetically modified strains) or methods/technologies to isolate and to purify those carotenoids. Table 2 summarises the patents focused on those subjects during the last 20 years.

## 7. Conclusions

On the basis of carotenoid production by haloarchaea in terms of quantity and variety, these microorganisms are revealed as good candidates to produce carotenoids at high-scale following cheap and quick culture and downstream processes. The most interesting carotenoids from a commercial point of view nowadays are not the major ones produced by haloarchaea as it can be concluded from the previous sections, with the exception of β-carotene, which is produced at significant concentration by several species. Since there are not studies on the potential benefits of the carotenoids produced by haloarchaea on human health reported in the scientific literature, more efforts should be made to properly address this question. Studies about carotenoid metabolism in haloarchaea are also required to provide further insights into the mechanisms controlling localized and context-specific carotenoid synthesis and degradation; such analysis would lead to a better understanding of the spatial distribution and function of different carotenoids and their derivatives in response to environmental and developmental signals. This knowledge may facilitate further progress in the field of carotenoid metabolic engineering in haloarchaea.

## References

[1] Zhang J., Sun Z., Sun P., Chen T., Chen F. (2014). Microalgal carotenoids: Beneficial effects and potential in human health. Food Funct..

[2] Fiedor J., Burda K. (2014). Potential role of carotenoids as antioxidants in human health and disease. Nutrients.

[3] Vílchez C., Forján E., Cuaresma M., Bédmar F., Garbayo I., Vega J.M. (2011). Marine carotenoids: Biological functions and commercial applications. Mar. Drugs.

[4] Nisar N., Li L., Lu S., Khin N.C., Pogson B.J. (2015). Carotenoid metabolism in plants. Mol. Plant.

[5] Yatsunami R., Ando A., Yang Y., Takaichi S., Kohno M., Matsumura Y., Ikeda H., Fukui T., Nakasone K., Fujita N. (2014). Identification of carotenoids from the extremely halophilic archaeon *Haloarcula japonica*. Front. Microbiol..

[6] Mata-Gómez L.C., Montañez J.C., Méndez-Zavala A., Aguilar C.N. (2014). Biotechnological production of carotenoids by yeasts: An overview. Microb. Cell Fact..

[7] Goodwin T.W., Britton G., Goodwin T.W. (1980). Distribution and analysis of carotenoids. Plant Pigments.

[8] Cunningham F.X., Gantt E. (1998). Genes and enzymes of carotenoid biosynthesis in plants. Annu. Rev. Plant Physiol. Plant Mol. Biol..

[9] Blanco A.M., Moreno J., del Campo J.A., Rivas J., Guerrero M.G. (2007). Outdoor cultivation of lutein-rich cells of *Muriellopsis* sp. in open ponds. Appl. Microbiol. Biotechnol..

[10] Nelis H.J., de Leenheer A.P. (1991). Microbial sources of carotenoid pigments used in foods and feeds. J. Appl. Bacteriol..

[11] Bourgaud F., Gravot A., Milesi S., Gontier E. (2001). Production of plant secondary metabolites: A historical perspective. Plant Sci..

[12] Olaizola M. (2003). Commercial development of microalgal biotechnology: From the test tube to the marketplace. Biomol. Eng..

[13] Lichtenthaler H.K., Buschmann C. (2001). Chlorophylls and Carotenoids: Measurement and Characterization by UV-VIS Spectroscopy. Curr. Protoc. Food Analyt.Chem..

[14] Azevedo-Meleiro C.H., Rodriguez-Amaya D.B. (2004). Confirmation of the identity of the carotenoids of tropical fruits by HPLC-DAD and HPLC-MS. J. Food Comp. Anal..

[15] Jaime L., Mendiola J., Herrero M., Soler-Rivas C., Santoyo S., Señorans F.J., Cifuentes A., Ibañez E. (2005). Separation and characterization of antioxidants from *Spirulina platensis* microalga combining pressurized liquid extraction, TLC, and HPLC-DAD. J. Sep. Sci..

[16] Hengartner U., Bernhard K., Meyer K., Englert G., Glinz E. (1992). Synthesis, isolation, and NMR-Spectroscopic characterization of Fourteen (*Z*)-Isomers of Lycopene and of come AcetylenicDidehydro- and Tetradehydrolycopenes. Helv. Chim. Acta.

[17] Britton G. (1995). Structure and properties of carotenoids in relation to function. FASEB J..

[18] Meléndez-Martínez A.J., Britton G., Vicario I.M., Heredia F.J. (2007). Relationship between the colour and the chemical structure of carotenoid pigments. Food Chem..

[19] De Lourdes Moreno M., Sánchez-Porro C., García M.T., Mellado E. (2012). Carotenoids’ production from halophilic bacteria. Methods Mol. Biol..

[20] Oren A. (2005). A hundred years of *Dunaliella* research: 1905–2005. Saline Syst..

[21] Oren A. (2014). The ecology of *Dunaliella* in high-salt environments. J. Biol. Res..

[22] Hosseini Tafreshi A., Shariati M. (2009). *Dunaliella* biotechnology: Methods and applications. J. Appl. Microbiol..

[23] Lamers P.P., Janssen M., de Vos R.C.H., Bino R.J., Wijffels R.H. (2008). Exploring and exploiting carotenoid accumulation in *Dunaliella salina* for cell-factory applications. Trends Biotechnol..

[24] Asker D., Ohta Y. (1999). Production of Canthaxanthin by Extremely Halophilic Bacteria. J. Biosci. Bioeng..

[25] Asker D., Awad T., Ohta T. (2002). Lipids of *Haloferax alexandrinus* Strain TMT. An Extremely Halophilic Canthaxanthin-Producing Archaeon. J. Biosci. Bioeng..

[26] Ronnekleiv M., Liaaen-Jensen S. (1995). Bacterial Carotenoids 53*, C_50_-Carotenoids 23; Carotenoids of *Haloferax volcanii versus* other Halophilic Bacteria. Biochem. Syst. Ecol..

[27] Gupta R.S., Naushad S., Baker S. (2015). Phylogenomic analyses and molecular signatures for the class *Halobacteria* and its two major clades: A proposal for division of the class *Halobacteria* into an emended order *Halobacteriales* and two new orders, *Haloferacales* ord nov and *Natrialbales* ord. nov., containing the novel families *Haloferacaceae* fam. nov. and *Natrialbaceae* fam. nov.. Int. J. Syst. Evol. Microbiol..

[28] Oren A. (2013). Life at high salt concentrations, intracellular KCl concentrations, and acidic proteomes. Front. Microbiol..

[29] Oren A. (2010). Industrial and environmental applications of halophilic microorganisms. Environ. Technol..

[30] Oren A. (2014). Halophilic archaea on Earth and in space: Growth and survival under extreme conditions. Philos. Trans. A Math. Phys. Eng. Sci..

[31] Schwieter U., Rüegg R., Isler O. (1966). Syntheses in the carotenoid series. 21. Synthesis of 2,2′-diketo-spirilloxanthin (P 518) and 2,2′-diketo-bacterioruberin. Helv. Chim. Acta.

[32] Kelly M., Jensen S.L. (1967). Bacterial carotenoids. XXVI. C_50_-carotenoids. 2. Bacterioruberin. Acta Chem. Scand..

[33] Rao A.V., Rao L.G. (2007). Carotenoids and human health. Pharmacol. Res..

[34] Del Campo J.A., García-González M., Guerrero M.G. (2007). Outdoor cultivation of microalgae for carotenoid production: Current state and perspectives. Appl. Microbiol. Biotechnol..

[35] Rivera S.M., Canela-Garayoa R. (2012). Analytical tools for the analysis of carotenoids in diverse materials. J. Chromatogr. A.

[36] Fassett R.G., Coombes J.S. (2012). Astaxanthin in cardiovascular health and disease. Molecules.

[37] Jehlička J., Oren A. (2013). Raman spectroscopy in halophile research. Front. Microbiol..

[38] Tanaka T., Shnimizu M., Moriwaki H. (2012). Cancer chemoprevention by carotenoids. Molecules.

[39] Higuera-Ciapara I., Félix-Valenzuela L., Goycoolea F.M. (2006). Astaxanthin: A review of its chemistry and applications. Crit. Rev. Food Sci. Nutr..

[40] Ambati R.R., Phang S.M., Ravi S., Aswathanarayana R.G. (2014). Astaxanthin: Sources, extraction, stability, biological activities and its commercial applications—A review. Mar. Drugs.

[41] Stutz H., Bresgen N., Eckl P.M. (2015). Analytical tools for the analysis of β-carotene and its degradation products. Free Radic. Res..

[42] Englert M., Hammann S., Vetter W. (2015). Isolation of β-carotene, α-carotene and lutein from carrots by countercurrent chromatography with the solvent system modifier benzotrifluoride. J. Chromatogr. A.

[43] Li Y., Liu S., Man Y., Li N., Zhou Y.U. (2015). Effects of vitamins E and C combined with β-carotene on cognitive function in the elderly. Exp. Ther. Med..

[44] Relevy N.Z., Harats D., Harari A., Ben-Amotz A., Bitzur R., Rühl R., Shaish A. (2015). Vitamin A-Deficient Diet Accelerated Atherogenesis in Apolipoprotein E(−/−) Mice and Dietary β-Carotene Prevents This Consequence. Biomed. Res. Int..

[45] Tanaka T., Makita H., Ohnishi M., Mori H., Satoh K., Hara A. (1995). Chemoprevention of rat oral carcinogenesis by naturally occurring xanthophylls, astaxanthin and canthaxanthin. Cancer Res..

[46] Surai P.F. (2012). The antioxidant properties of canthaxanthin and its potential effects in the poultry eggs and on embryonic development of the chick, Part 1. World Poult. Sci. J..

[47] Rostami F., Razavi S.H., Sepahi A.A., Gharibzahedi S.M. (2014). Canthaxanthin biosynthesis by *Dietzia natronolimnaea* HS-1: Effects of inoculation and aeration rate. Braz. J. Microbiol..

[48] Hojjati M., Razavi S.H., Rezaei K., Gilani K. (2014). Stabilization of canthaxanthin produced by *Dietzia natronolimnaea* HS-1 with spray drying microencapsulation. J. Food Sci. Technol..

[49] Heying E.K., Tanumihardjo J.P., Vasic V., Cook M., Palacios-Rojas N., Tanumihardjo S.A. (2014). Biofortified orange maize enhances β-cryptoxanthin concentrations in egg yolks of laying hens better than tangerine peel fortificant. J. Agric. Food Chem..

[50] Burri B.J. (2015). β-Cryptoxanthin as a source of vitamin A. J. Sci. Food Agric..

[51] Granado-Lorencio F., de Las Heras L., Millán C.S., Garcia-López F.J., Blanco-Navarro I., Pérez-Sacristán B., Domínguez G. (2014). β-Cryptoxanthin modulates the response to phytosterols in post-menopausal women carrying NPC1L1 L272L and ABCG8 A632 V polymorphisms: An exploratory study. Genes Nutr..

[52] Chisté R.C., Freitas M., Mercadante A.Z., Fernandes E. (2014). Carotenoids are effective inhibitors of *in vitro* hemolysis of human erythrocytes, as determined by a practical and optimized cellular antioxidant assay. J. Food Sci..

[53] Ghodratizadeh S., Kanbak G., Beyramzadeh M., Dikmen Z.G., Memarzadeh S., Habibian R. (2014). Effect of carotenoid β-cryptoxanthin on cellular and humoral immune response in rabbit. Vet. Res. Commun..

[54] Li D., Xiao Y., Zhang Z., Liu C. (2015). Light-induced oxidation and isomerization of all-*trans*-β-cryptoxanthin in a model system. J. Photochem. Photobiol. B Biol..

[55] Riccioni G., D’Orazio N., Franceschelli S., Speranza L. (2011). Marine carotenoids and cardiovascular risk markers. Mar. Drugs..

[56] Igielska-Kalwat J., Gościańska J., Nowak I. (2015). Carotenoids as natural antioxidants. Postepy Hig. Med. Dosw..

[57] Pirayesh Islamian J., Mehrali H. (2015). Lycopene as a carotenoid provides radioprotectant and antioxidant effects by quenching radiation-induced free radical singlet oxygen: An overview. Cell J..

[58] Naziri D., Hamidi M., Hassanzadeh S., Tarhriz V., Maleki Zanjani B., Nazemyieh H., Hejazi M.A., Hejazi M.S. (2014). Analysis of Carotenoid Production by *Halorubrum.* sp. TBZ126: An Extremely Halophilic Archeon from Urmia Lake. Adv. Pharm. Bull..

[59] Flaks B., Bresloff P. (1966). Some observations on the fine structure of the lutein cells of X-irradiated rat ovary. J. Cell Biol..

[60] Altemimi A., Lightfoot D.A., Kinsel M., Watson D.G. (2015). Employing Response Surface Methodology for the Optimization of Ultrasound Assisted Extraction of Lutein and β-Carotene from Spinach. Molecules.

[61] Huang Y.M., Dou H.L., Huang F.F., Xu X.R., Zou Z.Y., Lin X.M. (2015). Effect of supplemental lutein and zeaxanthin on serum, macular pigmentation, and visual performance in patients with early age-related macular degeneration. Biomed. Res. Int..

[62] Costa S., Giannantonio C., Romagnoli C., Barone G., Gervasoni J., Perri A., Zecca E. (2015). Lutein and zeaxanthin concentrations in formula and human milk samples from Italian mothers. Eur. J. Clin. Nutr..

[63] Li X.R., Tian G.Q., Shen H.J., Liu J.Z. (2015). Metabolic engineering of *Escherichia coli* to produce zeaxanthin. J. Ind. Microbiol. Biotechnol..

[64] Yamamoto H.Y., Chang J.L., Aihara M.S. (1967). Light-induced interconversion of violaxanthin and zeaxanthin in New Zealand spinach-leaf segments. Biochim. Biophys. Acta.

[65] Yamamoto H.Y., Kamite L., Wang Y.Y. (1972). An Ascorbate-induced Absorbance Change in Chloroplasts from Violaxanthin De-epoxidation. Plant Physiol..

[66] Sapozhnikov D.I. (1973). Investigation on the violaxanthin cycle. Pure Appl. Chem..

[67] Soontornchaiboon W., Joo S.S., Kim S.M. (2012). Anti-inflammatory effects of violaxanthin isolated from microalga *Chlorella ellipsoidea* in RAW 264.7 macrophages. Biol. Pharm Bull..

[68] Hallin E.I., Guo K., Åkerlund H.E. (2015). Violaxanthin de-epoxidase disulphides and their role in activity and thermal stability. Photosynth. Res..

[69] Burton G.W., Foster D.O., Perly B., Slater T.F., Smith I.C., Ingold K.U. (1985). Biological antioxidants. Philos. Trans. R. Soc. Lond. B Biol. Sci..

[70] Gammone M.A., Riccioni G., D’Orazio N. (2015). Carotenoids: Potential allies of cardiovascular health?. Food Nutr. Res..

[71] LaFountain A.M., Prum R.O., Frank H.A. (2015). Diversity, physiology, and evolution of avian plumage carotenoids and the role of carotenoid-protein interactions in plumage colour appearance. Arch. Biochem. Biophys..

[72] Namitha K.K., Negi P.S. (2010). Chemistry and biotechnology of carotenoids. Crit. Rev. Food Sci. Nutr..

[73] Zile M.H. (1998). Vitamin A and embryonic development: An overview. J. Nutr..

[74] Kaulmann A., Bohn T. (2014). Carotenoids, inflammation, and oxidative stress—Implications of cellular signaling pathways and relation to chronic disease prevention. Nutr. Res..

[75] Gupta C., Prakash D. (2014). Phytonutrients as therapeutic agents. J. Complement. Integr. Med..

[76] Ascenso A., Ribeiro H., Marques H.C., Oliveira H., Santos C., Simões S. (2014). Chemoprevention of photocarcinogenesis by lycopene. Exp. Dermatol..

[77] Amundsen C.R., Nordeide J.T., Gjøen H.M., Larsen B., Egeland E.S. (2015). Conspicuous carotenoid-based pelvic spine ornament in three-spined stickleback populations—Occurrence and inheritance. Peer J..

[78] Jehlicka J., Edwards H.G., Oren A. (2013). Bacterioruberin and salinixanthin carotenoids of extremely halophilic Archaea and Bacteria: A Raman spectroscopic study. Spectrochim. Acta A Mol. Biomol. Spectrosc..

[79] Mandelli F., Miranda V.S., Rodrigues E., Mercadante A.Z. (2012). Identification of carotenoids with high antioxidant capacity produced by extremophile microorganisms. World J. Microbiol. Biotechnol..

[80] Othman R., Mohd Zaifuddin F.A., Hassan N.M. (2014). Carotenoid biosynthesis regulatory mechanisms in plants. J. Oleo Sci..

[81] Palczewski G., Amengual J., Hoppel C.L., von Lintig J. (2014). Evidence for compartmentalization of mammalian carotenoid metabolism. FASEB J..

[82] Giuliano G. (2014). Plant carotenoids: Genomics meets multi-gene engineering. Curr. Opin. Plant Biol..

[83] Parker R.S. (1996). Absorption, metabolism, and transport of carotenoids. FASEB J..

[84] Reboul E., Borel P. (2011). Proteins involved in uptake, intracellular transport and basolateral secretion of fat-soluble vitamins and carotenoids by mammalian enterocytes. Prog. Lipid Res..

[85] Desmarais D., Jablonski P.E., Fedarko N.S., Roberts M.F. (1997). 2-Sulfotrehalose, a novel osmolyte in haloalkaliphilic archaea. J. Bacteriol..

[86] Madern D., Camacho M., Rodríguez-Arnedo A., Bonete M.J., Zaccai G. (2004). Salt-dependent studies of NADP-dependent isocitrate dehydrogenase from the halophilic archaeon *Haloferax volcanii*. Extremophiles.

[87] Bonete M.J., Martínez-Espinosa R.M., Ventosa A., Oren A. (2011). Enzymes from Halophilic Archaea: Open Questions. Halophiles and Hypersaline Environments: Current Research and Future Trends.

[88] Kushwaha S.C., Kramer J.K., Kates M. (1975). Isolation and characterization of C_50_-carotenoid pigments and other polar isoprenoids from *Halobacterium cutirubrum*. Biochim. Biophys. Acta.

[89] Bidle K.A., Hanson T.E., Howell K., Nannen J. (2007). HMG-CoA reductase is regulated by salinity at the level of transcription in *Haloferax volcanii*. Extremophiles.

[90] Oren A., Gurevich P. (1995). Dynamics of a bloom of halophilic archaea in the Dead Sea. Hydrobiologia.

[91] Asker D., Awad T., Ohta Y. (2002). Lipids of *Haloferax. alexandrinus* strain TM^T^: An extremely halophilic canthaxanthin-producing archaeon. J. Biosci. Bioeng..

[92] Marshall C.P., Leuko S., Coyle C.M., Walter M.R., Burns B.P., Neilan B.A. (2007). Carotenoid analysis of halophilic archaea by resonance Raman spectroscopy. Astrobiology.

[93] Jehlička J., Edwards H.G., Oren A. (2014). Raman spectroscopy of microbial pigments. Appl. Environ. Microbiol..

[94] Lobasso S., Lopalco P., Mascolo G., Corcelli A. (2008). Lipids of the ultra-thin square halophilic archaeon *Haloquadratum walsbyi*. Archaea.

[95] Kushwaha S.C., Kates M., Porter J.W. (1976). Enzymatic synthesis of C_40_ carotenes by cell-free preparation from *Halobacterium cutirubrum*. Can. J. Biochem..

[96] Kushwaha S.C., Kates M. (1976). Effect of nicotine on biosynthesis of C_50_ carotenoids in *Halobacterium cutirubrum*. Can. J. Biochem..

[97] Kushwaha S.C., Kates M. (1979). Effect of glycerol on carotenogenesis in the extreme halophile, *Halobacterium cutirubrum*. Can. J. Microbiol..

[98] Peck R.F., Echavarri-Erasun C., Johnson E.A., Ng W.V., Kennedy S.P., Hood L., DasSarma S., Krebs M.P. (2001). *brp* and *blh* are required for synthesis of the retinal cofactor of bacteriorhodopsin in *Halobacterium salinarum*. J. Biol. Chem..

[99] Dassarma S., Kennedy S.P., Berquist B., Victor N.W., Baliga N.S., Spudich J.L., Krebs M.P., Eisen J.A., Johnson C.H., Hood L. (2001). Genomic perspective on the photobiology of *Halobacterium* species NRC-1, a phototrophic, phototactic, and UV-tolerant haloarchaeon. Photosynth. Res..

[100] Falb M., Müller K., Königsmaier L., Oberwinkler T., Horn P., von Gronau S., Gonzalez O., Pfeiffer F., Bornberg-Bauer E., Oesterhelt D. (2008). Metabolism of halophilic archaea. Extremophiles.

[101] Oesterhelt D. (1976). Bacteriorhodopsin as an example of a light-driven proton pump. Angew. Chem. Int. Ed. Engl..

[102] Sumper M., Reitmeier H., Oesterhelt D. (1976). Biosynthesis of the purple membrane of halobacteria. Angew. Chem. Int. Ed. Engl..

[103] Yang Y., Yatsunami R., Ando A., Miyoko N., Fukui T., Takaichi S., Nakamura S. (2015). Complete Biosynthetic Pathway of the C_50_ Carotenoid Bacterioruberin from Lycopene in the extremely halophilic archaeon *Haloarcula japonica*. J. Bacteriol..

[104] Rodrigo-Baños M., Garbayo I., Vilchez C., Bonete M.J., Martínez.Espinosa R.M. Genomic analysis of the biosynthesis of isoprenoids in *Haloferax* genus, to be submitted for publication.

[105] Fang C.J., Ku K.L., Lee M.H., Su N.W. (2010). Influence of nutritive factors on C_50_ carotenoids production by *Haloferax mediterranei* ATCC 33500 with two-stage cultivation. Bioresour. Technol..

[106] Dundas I.D., Larsen H. (1963). A study on the killing by light of photosensitized cells of *Halobacterium salinarium*. Arch. Mikrobiol..

[107] Shahmohammadi H.R., Asgarani E., Terato H., Saito T., Ohyama Y., Gekko K., Yamamoto O., Ide H. (1998). Protective roles of bacterioruberin and intracellular KCl in the resistance of *Halobacterium salinarium* against DNA-damaging agents. J. Radiat. Res..

[108] Kelly M., Norgard S., Liaaen-Jensen S. (1970). Bacterial carotenoids. 31. C_50_-carotenoids 5. Carotenoids of *Halobacterium. salinarium*, especially bacterioruberin. Acta Chem. Scand..

[109] Becher B.M., Cassim J.Y. (1975). Improved isolation procedures for the purple membrane of *Halobacterium halobium*. Prep. Biochem..

[110] Kushwaha S.C., Kates M. (1979). Studies of the biosynthesis of C_50_ carotenoids in *Halobacterium cutirubrum*. Can. J. Microbiol..

[111] Shand R.F., Betlach M.C. (1991). Expression of the bop gene cluster of *Halobacterium halobium* is induced by low oxygen tension and by light. J. Bacteriol..

[112] El-Sayed W.S., Takaichi S., Saida H., Kamekura M., Abu-Shady M., Seki H., Kuwabara T. (2002). Effects of light and low oxygen tension on pigment biosynthesis in *Halobacterium salinarum*, revealed by a novel method to quantify both retinal and carotenoids. Plant Cell Physiol..

[113] D’Souza S.E., Altekar W., D’Souza S.F. (1997). Adaptive response of *Haloferax mediterranei* to low concentrations of NaCl (<20%) in the growth medium. Arch. Microbiol..

[114] Raghavan T.M., Furtado I. (2005). Expression of carotenoid pigments of haloarchaeal cultures exposed to aniline. Environ. Toxicol..

[115] Raghavan T., Furtado I. (2004). Occurrence of extremely halophilic Archaea in sediments from the continental shelf of west coast of India. Curr. Sci..

[116] Hamidi M., Abdin M.Z., Nazemyieh H., Hejazi M.A., Hejazi M.S. (2014). Optimization of Total Carotenoid Production by *Halorubrum* sp. TBZ126 using response surface methodology. J. Microb. Biochem. Technol..

[117] Fendrihan S., Musso M., Stan-Lotter H. (2009). Raman spectroscopy as a potential method for the detection of extremely halophilic archaea embedded in halite in terrestrial and possibly extraterrestrial samples. J. Raman Spectrosc..

[118] Miller N.J., Sampson J., Candeias L.P., Bramley P.M., Rice-Evans C.A. (1996). Antioxidant activities of carotenes and xanthophylls. FEBS Lett..

[119] Albrecht M., Takaichi S., Steiger S., Wang Z.Y., Sandmann G. (2000). Novel hydroxycarotenoids with improved antioxidative properties produced by gene combination in *Escherichia coli*. Nat. Biotechnol..

[120] Tian B., Xu Z., Sun Z., Lin J., Hua Y. (2007). Evaluation of the antioxidant effects of carotenoids from *Deinococcus radiodurans* through targeted mutagenesis, chemiluminescence, and DNA damage analyses. Biochim. Biophys. Acta.

[121] Saito T., Miyabe Y., Ide H., Yamamoto O. (1997). Hydroxyl radical scavenging ability of bacterioruberin. Radiat. Phys. Chem..

[122] Kottemann M., Kish A., Iloanusi C., Bjork S., DiRuggiero J. (2005). Physiological responses of the halophilic archaeon *Halobacterium* sp. strain NRC1 to desiccation and gamma irradiation. Extremophiles.

[123] Lazrk T., Wolff G., Albrecht A.M., Nakatani Y., Ourisson G., Kates M. (1988). Bacterioruberins reinforce reconstituted halobacterium lipid-membranes. Biochim. Biophys. Acta.

[124] Cao Z., Ding X., Peng B., Zhao Y., Ding J., Watts A., Zhao X. (2015). Novel expression and characterization of a light driven proton pump archaerhodopsin-4 in a *Halobacterium salinarum* strain. Biochim. Biophys. Acta.

[125] Feng J., Liu H.C., Chu J.F., Zhou P.J., Tang J.A., Liu S.J. (2006). Genetic cloning and functional expression in *Escherichia coli* of an archaerhodopsin gene from *Halorubrum xinjiangense*. Extremophiles.

[126] Li Q., Sun Q., Zhao W., Wang H., Xu D. (2000). Newly isolated archaerhodopsin from a strain of Chinese halobacteria and its proton pumping behavior. Biochim. Biophys. Acta.

[127] Yoshimura K., Kouyama T. (2008). Structural role of bacterioruberin in the trimeric structure of archaerhodopsin-2. J. Mol. Biol..

[128] Sasaki T., Razak N.W., Kato N., Mukai Y. (2012). Characteristics of halorhodopsin-bacterioruberin complex from *Natronomonas pharaonis* membrane in the solubilized system. Biochemistry.

[129] Google (Key words: caroten and haloarchaea). https://www.google.es/?tbm=pts&gws_rd=cr,ssl&ei=md8wVcesMYyCPeKdgdgC#tbm=pts&q=caroten+%26+haloarchaea+patents.

[130] Pantentscope (Key words: halobacteria, carotenoids and haloarchaea). https://patentscope.wipo.int/search/en/result.jsf.

[131] Oficina Española de patentes y marcas - Invenciones. http://www.oepm.es/es/invenciones/resultados.html?field=TITU_RESU&bases=0&keyword=carotenoid.

[132] World Intellectual Property Organization Global Brand Database. http://www.wipo.int/branddb/en.

[133] Japan Platform for Patent information website. https://www.j-platpat.inpit.go.jp/web/all/top/BTmTopEnglishPage.

[134] Espacenet Patent search. http://worldwide.espacenet.com/?locale=en_EP.

[135] European patent register. https://register.epo.org/regviewer.

[136] Markets and markets website—New market reports. http://www.marketsandmarkets.com/search.asp?Search=carotenoid&x=0&y=0.

[137] Yachai M. (2009). Carotenoid Production by Halophilic Archaea and Its Applications. Ph.D. Thesis.

[138] Varela J.C., Pereira H., Vila M., León R. (2015). Production of carotenoids by microalgae: Achievements and challenges. Photosynth. Res..

[139] Norsker N., Barbosa M., Vermue M., Wijffels R. (2001). Microalgal production: A close look at the economics. Biotechnol. Adv..

[140] Wichuk K., Brynjolfsson S., Fu W. (2014). Biotechnological production of value-added carotenoids from microalgae: Emerging technology and prospects. Bioengineered.

[141] Zeng C., Zhu J.C., Liu Y., Yang Y., Zhu J.Y., Huang Y.P., Shen P. (2006). Investigation of the influence of NaCl concentration on *Halobacterium salinarum* growth. J. Therm. Anal. Calorim..

[142] Mata T., Martins A., Caetano N. (2010). Microalgae for biodiesel production and other applications: A review. Renew. Sustain. Energy Rev..

[143] Abbes M., Baati H., Guermazi S., Messina C., Santulli A., Gharsallah N., Ammar E. (2013). Biological properties of carotenoids extracted from *Halobacterium halobium* isolated from a Tunisian solar saltern. BMC Complement. Altern. Med..

[144] Sikkandar S., Murugan K., Al-Sohaibani S., Rayappan F., Nair A., Tilton F. (2013). Halophilic bacteria-A potent source of carotenoids with antioxidant and anticancer potentials. J. Pure Appl. Microbiol..

